# The Dysferlin Transcript Containing the Alternative Exon 40a is Essential for Myocyte Functions

**DOI:** 10.3389/fcell.2021.754555

**Published:** 2021-11-23

**Authors:** Océane Ballouhey, Sébastien Courrier, Virginie Kergourlay, Svetlana Gorokhova, Mathieu Cerino, Martin Krahn, Nicolas Lévy, Marc Bartoli

**Affiliations:** ^1^ INSERM, MMG, U1251, Aix Marseille University, Marseille, France; ^2^ AP-HM, Département de Génétique Médicale, Hôpital d’Enfants de la Timone, Marseille, France; ^3^ GIPTIS, Genetics Institute for Patients Therapies Innovation and Science, Marseille, France

**Keywords:** membrane, calpain (CAPN), dysferlin, dysferlinopathies, neuromuscular disease

## Abstract

Dysferlinopathies are a group of muscular dystrophies caused by recessive mutations in the DYSF gene encoding the dysferlin protein. Dysferlin is a transmembrane protein involved in several muscle functions like T-tubule maintenance and membrane repair. In 2009, a study showed the existence of fourteen dysferlin transcripts generated from alternative splicing. We were interested in dysferlin transcripts containing the exon 40a, and among them the transcript 11 which contains all the canonical exons and exon 40a. This alternative exon encodes a protein region that is cleaved by calpains during the muscle membrane repair mechanism. Firstly, we tested the impact of mutations in exon 40a on its cleavability by calpains. We showed that the peptide encoded by the exon 40a domain is resistant to mutations and that calpains cleaved dysferlin in the first part of DYSF exon 40a. To further explore the implication of this transcript in cell functions, we performed membrane repair, osmotic shock, and transferrin assay. Our results indicated that dysferlin transcript 11 is a key factor in the membrane repair process. Moreover, dysferlin transcript 11 participates in other cell functions such as membrane protection and vesicle trafficking. These results support the need to restore the dysferlin transcript containing the alternative exon 40a in patients affected with dysferlinopathy.

## Introduction

Dysferlinopathies are disabling muscle disorders caused by recessive mutations in the DYSF gene (MIM# 603009, 2p13, GenBank NM_003494.3) encoding the dysferlin protein. At the clinical level, dysferlinopathies exist in two different forms of muscular dystrophies namely LGMD2B/R2 (LGMD2B; MIM# 253601) and Miyoshi Myopathy (MM; MIM# 254130) (Bashir et al., 1998). The main muscle groups affected at onset are proximal muscles in LGMD2B and distal muscles in MM (Liu et al., 1998). Highly elevated serum levels of creatine kinase, severe muscle inflammation and muscle weakness are characteristic of dysferlinopathies patients. To date, there is no cure for these patients but several therapeutic strategies are currently under development like gene therapy.

Dysferlin is a transmembrane protein enriched in muscle, composed of C2 domains and is involved in various functions of the muscular cells: protein vesicle trafficking and fusion (Lek et al., 2012), T tubule formation, membrane repair (Bansal et al., 2003; Glover and Brown 2007; Han and Campbell 2007; Krahn et al., 2010a; Lek et al., 2013; Defour et al., 2014; McDade et al., 2014; Redpath et al., 2014) and myoblast/myotube membrane fusion (de Luna et al., 2006; Demonbreun et al., 2011; Balasubramanian et al., 2014). This membrane repair mechanism relies on different pathways, such as the lysosomal and the endosomal pathways, both involving dysferlin activity (Defour et al., 2014). Studies in recent years have led to a better understanding of the membrane repair process involving dysferlin by identifying many proteins involved in this process such as caveolin-3, MG53, AHNAK, annexins and others. (Matsuda et al., 2001; Lennon et al., 2003; de Luna et al., 2006; Waddell et al., 2011; Blandin et al., 2013). The cytoskeleton is also involved in this process, for instance, the patch formation necessary for membrane repair requires Kif5B for microtubule-based transport of dysferlin-containing vesicles and membrane-vesicle fusion (McDade et al., 2014; McDade and Michele, 2014). It has been shown that membrane injury induced a calcium influx which led to calpain activation, then, calpains cleaved dysferlin in the protein region encoded by the alternative exon 40a, releasing a C-terminal fragment named mini-dysferlin which has a major role in membrane repair mechanism (Lek et al., 2013; Redpath et al., 2014).

In 2009, Pramono and colleagues demonstrated the presence of fourteen dysferlin transcripts from alternative splicing involving exons 5a, 17 and 40a with seven transcript variants generated under the promoter of DYSF (GenBank AF075575) and seven transcript variants generated under the promoter of DYSF_v1 (GenBank DQ267935) (Pramono et al., 2009). Among these fourteen transcripts, six of them contain the alternative exon 40a and are expressed at varying rates in skeletal muscle (from 0 to 4%). Mutation screening in the three known dysferlin alternative exons, DYSF-v1, 5a and 40a, has been previously studied and no hot spot has been found (Krahn et al., 2010b).

Nonetheless, given the functional importance of the cleavage site encoded by exon 40a, transcripts containing this alternative exon seem to be important for the functions of dysferlin. To further explore this hypothesis, we performed a functional study to discover the implication of this transcript in cell functions. We also tried to identify the localization of the calpain cleavage site.

In this work we have shown, *in vitro,* that the exon 40a calpain cleavage site was located in the first part of this exon, and that transcripts containing this alternative exon are key factors in the membrane repair process. Furthermore, dysferlin transcript 11 may participate in other cell functions like membrane protection and protein vesicle trafficking. These results recommend that the dysferlin transcript containing alternative exon 40a could be included in vectors used in gene transfer experiments to treat patients with dysferlinopathy.

## Materials and Methods

### Cell Cultures

Wild type (WT) (C25) and DYSF-null (AB320) human myoblasts cells lines were given by Vincent Mouly from the Myology Research Center (Paris, France). Cells were grown in a humidified environment at 37°C and 5% CO2 in Dulbecco’s modified Eagle’s medium, supplemented with 15% medium 199, 15% fetal bovine serum, 25 µg/ml fetuin, 5 ng/ml hEGF, 0.5 ng/ml bFGF, 5 µg/ml insulin and 0.2 µg/ml dexamethasone.

C2C12 murine myoblasts and HEK cells lines were bought at ATCC. Cells were grown in a humidified environment at 37°C and 5% CO2 in Dulbecco’s modified Eagle’s medium (ThermoFisher), supplemented with 20% fetal bovine serum and 100 µg/ml antibiotic antimycotic (GE Healthcare, ref P11-002).

### Plasmid Construction

The GFP-dysferlin1 plasmid was a generous gift from Dr. Kate Bushby, it contains the transcript of the main isoform of dysferlin (exon 1–55). The mCherry-dysferlin1 plasmid was generated by GFP switching to mCherry (Shaner et al., 2004) using the EcoRI and KpnI restriction enzymatic sites.

The coding sequence of GFP-dysferlin11 was generated by Cliniscience by inserting the alternative exon 40a between exon 40 and exon 41 into the GFP-dysferlin1 plasmid to reproduce the transcript 11 of the dysferlin. The mCherry-dysferlin11 plasmid was generated by GFP switching to mCherry using the EcoRI and KpnI restriction enzymatic sites. Dysferlin11 constructs to identify the cleavage site by calpain in DYSF exon 40a were created by PCR fusion (Table 1).

**TABLE 1:** Primers used to create dysferlin11 constructs by PCR fusion.

pcDNA Dysf11 delN	Forward	GCC​CCT​CAT​CCC​CAT​CCA​GAA​CAC​GGC​TTC​TCC​TCC​ATC​CAG​TCC​TC
	Reverse	GAT​GGA​GGA​GAA​GCC​GTG​TTC​TGG​ATG​GGG​ATG​AGG​GGC​TCC​TTG​TCA​TC
pcDNA Dysf11 delC	Forward	GTC​GAG​CTT​GGC​CCC​CAC​TGA​GGA​AGA​GTT​CAT​CGA​TTG​GTG​GAG​C
	Reverse	CCA​ATC​GAT​GAA​CTC​TTC​CTC​AGT​GGG​GGC​CAA​GCT​CGA​CAG​ACC​GTC
pcDNA Dysf11 delN1	Forward	CCT​CAT​CCC​CAT​CCA​GAG​CTT​GGC​CCC​CAC​TAA​CAC​GGC​TTC​TCC
	Reverse	GTT​AGT​GGG​GGC​CAA​GCT​CTG​GAT​GGG​GAT​GAG​GGG​CTC​CTT​GTC​ATC
pcDNA Dysf11 delN2	Forward	GCT​TGC​AGA​CGG​TCT​GTC​GAA​CAC​GGC​TTC​TCC​TCC​ATC​CAG​TCC
	Reverse	GGA​GGA​GAA​GCC​GTG​TTC​GAC​AGA​CCG​TCT​GCA​AGC​TGG​ATG​GGG

### Immunofluorescence

C2C12 myoblasts were grown on Lab-TEK II™ (Fisher Scientific) and transfected using Lipofectamine 2000 (Invitrogen) per the manufacturer’s directions. After 24 h, cells were injured with 0.1 mg glass beads. Glass beads were deposited inside the wells and wells were orbitally shaken to roll the beads onto the cells. Cells were incubated at 37°C for 5 min, fixed with 4% paraformaldehyde for 10 min and then washed in PBS. Cells were then incubated for 10 min with a permeabilization solution (200 μL of PBS 1X +0.5% triton X-100 + protease inhibitors cocktail) (Roche). From there, cells were exposed to a blocking buffer (PBS+ 1% BSA + protease inhibitors cocktail) for 30 min. The primary antibody (Hamlet-1, dysferlin, 1:40, Abcam 75571) was applied in blocking buffer for 3 h at room temperature, followed by a wash in PBS and 1 h of contact with the secondary antibody (Dylight 550 donkey anti-mouse IgG, 1:100, Abcam 96876) in blocking buffer. After washing in PBS, Lab-TEK II™ were mounted with Vectashield-Dapi 25 ng/ml and kept at 4°C until pictures were taken. The observation was performed using a Zeiss Axio Imager Z2 microscope (×40 objective), and images were processed with ZEN software and/or ImageJ software.

### Cell Scratch Injury, SDS-PAGE and Western Blot

HEK cells were plated at 70% confluence in a 6-well plate (VWR) and transfected using Lipofectamine 2000 (Invitrogen) per the manufacturer’s directions. After 24 h, cells were injured with 30 µM ionomycin and cell scrappers, then they were pelleted at 300 g for 5 min and the cell pellet was solubilized in RIPA buffer (Life technologies) and protease inhibitor cocktail (Life technologies). Samples were separated by SDS-PAGE on 3–8% NuPAGE Tris-Acetate gels (Life technologies) using Chameleon Duo as a size marker and transferred onto nitrocellulose membranes (at 100 V for 3 h at 4°C). Membranes were blocked using fluorescent WB blocking buffer (Tebu-bio) in TBS 1X for 1 h at room temperature. Primary antibodies (Hamlet-1, 1:150, Abcam 75571) were then diluted in blocking buffer and incubated overnight at 4°C. After washing in TBS-T, membranes were then incubated with secondary antibodies (IRDye 800CW Donkey anti-mouse, Li-Cor, ref 926-32212), which were diluted 1:10000 in blocking buffer for 45 min at room temperature. The membranes were washed in TBS-T and developed using NIR Fluorescence LI-COR. Actin (1:5,000, Merck MA1501r) was detected using a dilution of 1:10000 of secondary antibodies (IRDye 680RD Donkey anti-mouse, Li-Cor, ref 925-68072).

### Membrane Repair Assay

WT (C25) and DYSF-null (AB320) human myoblasts were plated at 50% confluence on a 6-well plate (VWR) and transfected using Lipofectamine 2000 (Invitrogen) per manufacturer’s directions. After 24 h, cells were visualized in the presence of Ca2+ (1 mM) and membrane-impermeable dye FM 1-43 (2.5 µM, Molecular Probes) with a confocal microscope (LSM ×800, ×63 objective, ZEISS). Membrane damage was applied to myoblasts using a UV laser to irradiate a 0.33 µm2 area at maximum power for 15 sec at t = 10 sec. Images were captured every second for 5 min, and the mean fluorescence intensity of FM1-43 was measured on a 13.5 µm2 area around the damage with the Zeiss LSM 800 imaging software.

### Osmotic Shock Assay

WT (C25) and DYSF-null (AB320) human myoblasts were plated at 70% confluence on a 12-well plate (VWR) and transfected using Lipofectamine 2000 (Invitrogen) per manufacturer’s directions. After 24 h, myoblasts were washed with distilled water and hypo-osmotic shock was performed by incubating cells with distilled water. Cell fluorescence was followed with Fast imaging observer (Axio observer, Z1/×7, ×10 objective, ZEISS). Images were captured every minute for 30 min.

### Transferrin Uptakes and Recycling Assays

WT (C25) and DYSF-null (AB320) human myoblasts were plated at 70% confluence on Lab-TEK II™ (Fisher Scientific) and transfected using Lipofectamine 2000 (Invitrogen) per manufacturer’s directions. After 24 h, myoblasts were incubated in Dulbecco’s modified Eagle’s medium, supplemented with 1% L-glutamine and 0.5% bovine serum albumin at 37°C and 5% CO2 for 30 min. Then, cells were incubated in the same medium with 25 µg/ml transferrin from human serum (coupled with Alexa Fluor 488, Thermo Scientific) at 37°C and 5% CO2 for 30 min. For transferrin recycling assays, cells were washed in cold PBS and incubated in Dulbecco’s modified Eagle’s medium, supplemented with 15% medium 199, 15% fetal bovine serum, 25 µg/ml fetuin, 5 ng/ml hEGF, 0.5 ng/ml bFGF, 5 µg/ml insulin and 0.2 µg/ml dexamethasone. Then, myoblasts were washed in PBS and incubated in stripping buffer (NaCl, acid acetic) for a few seconds. After that, cells were washed in PBS, fixed with 4% paraformaldehyde for 10 min and washed again in PBS. Lab-TEK II™ were mounted with Vectashield-Dapi 25 ng/ml and kept at 4°C until pictures were taken. The observation was performed using a Zeiss Axio Imager Z2 microscope (×20 objective), and images were processed with ZEN software and/or ImageJ software.

### Statistical Analysis

Individual means were compared using the non-parametric Mann-Whitney test. The powers of the tests were strictly superior at 92%. Differences were statistically significant or very significant if *p* < 0.05 (*) or *p* < 0.01 (**), respectively.

## Results

### Glass Beads Injury Activates Calpain-Mediated Cleavage of Dysferlin Exon 40a.

**FIGURE 1:**
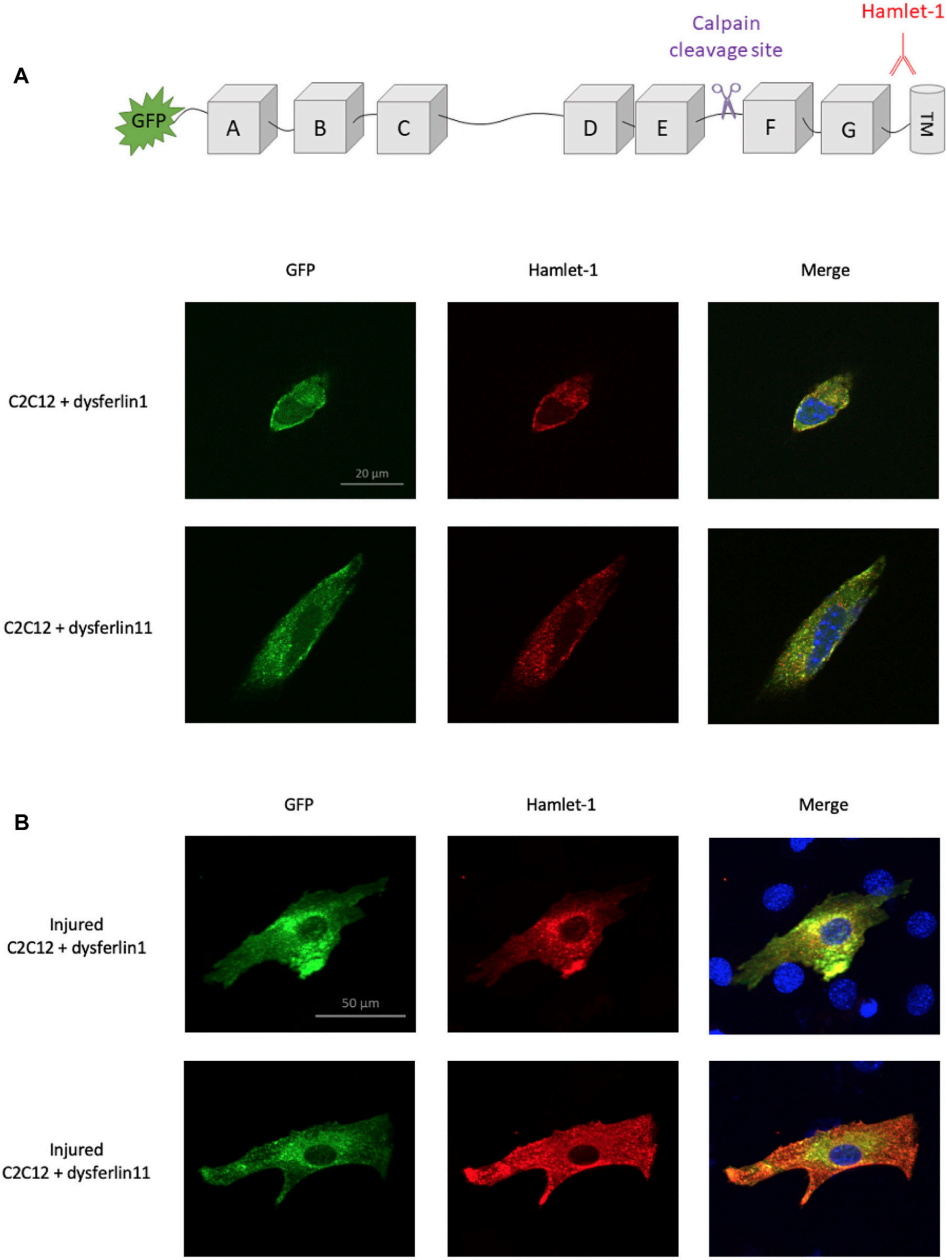
Localization difference between N-ter and C-ter part of dysferlin transcript 11. Dysferlin labeling performed on transfected and uninjured **(A)** or injured **(B)** C2C12 murine myoblasts (C2C12 + GFP-dysferlin1 and C2C12 + GFP-dysferlin11) by immunofluorescence with dysferlin antibody (Hamlet-1, red). GFP was used to observe the N-terminal part of dysferlin and the Hamlet-1 antibody to detect the C-terminal part of dysferlin, as shown in the cartoon. DAPI was used as a nucleus marker (blue). All images were captured by an ApoTome microscope (Zeiss). Scale bar, 100 µM.

In 2013, Lek and colleagues demonstrate that membrane injury causes calcium influx at injury sites which induces local activation of calpains (Lek et al., 2013). This activation led to dysferlin cleavage by calpains within exon 40a, releasing a C-terminal fragment named dysferlinC72 which is recruited to membrane injury sites. In this context, we transfected dysferlin transcript 1 (major transcript without exon 40a) and dysferlin transcript 11 (transcript containing exon 40a) in murine C2C12 cells to monitor dysferlin location after membrane injury. To this end, we performed glass beads injury and immunofluorescence on transfected C2C12 cells, uninjured (Figure 1A) or injured (Figure 1B). Dysferlin has been detected with the fused GFP protein in the N-terminal domain and with Hamlet-1 antibody which recognizes a C-terminal epitope, as shown in the sketch in Figure 1. Uninjured C2C12 transfected with dysferlin transcripts 1 or 11 show a similar location for the N-terminal and C-terminal parts of dysferlin. The same observation was made on injured C2C12 transfected with dysferlin transcript 1. In contrast, injured C2C12 transfected with dysferlin transcript 11 showed a strong membrane presence for the dysferlin C-terminal part and diffuse staining in the cytoplasm for the N-terminal part labelled by GFP. This result confirms the fact that the C-terminal fragment of dysferlin is recruited to the membrane injury site.

### Calpain Cleavage Site in DYSF Exon 40a

**FIGURE 2:**
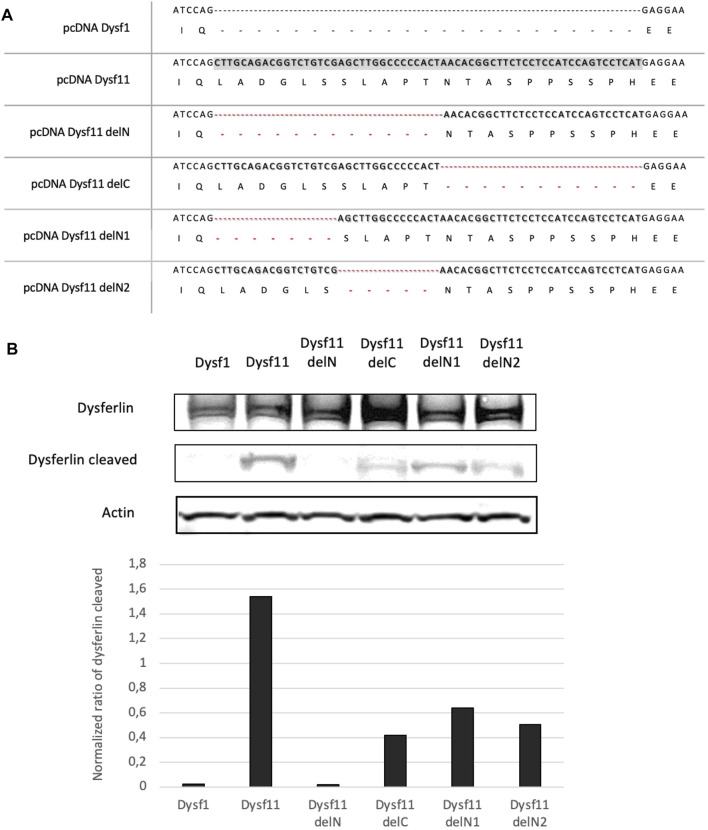
The N-terminal part of DYSF exon 40a is essential for calpain cleavage. **(A)** A Summary of dysferlin11 constructs created to identify calpain cleavage site in DYSF exon 40a. Constructs were built by PCR fusion. (B) Western-Blot performed with proteins from HEK cells transfected with dysferlin constructs and injured by ionomycin and cells scrapers 24 h post-transfection. Hamlet-1 was used to detect dysferlin and the cleaved dysferlin. Actin staining was used for normalization.

**FIGURE 3:**
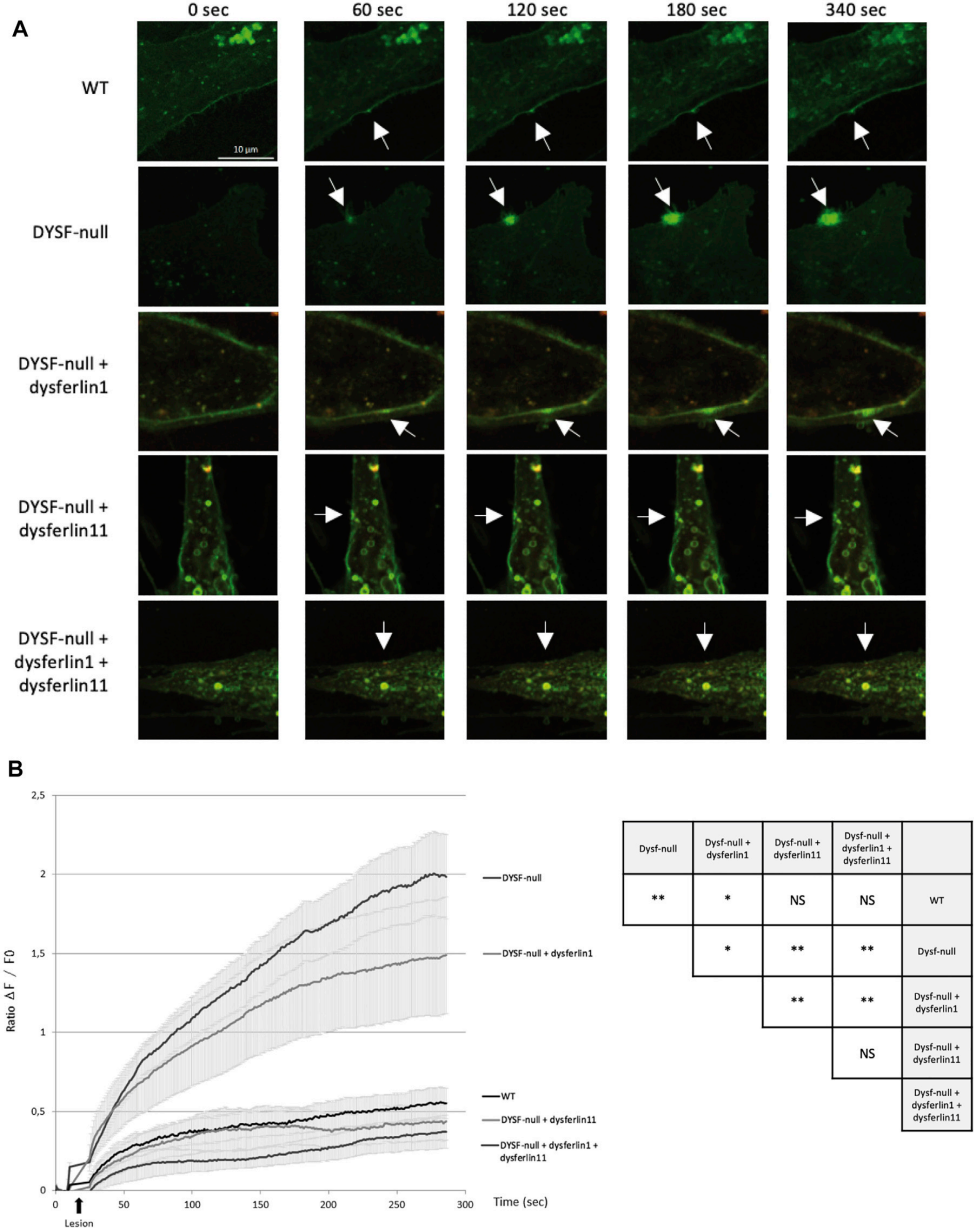
Dysferlin 11 transcript is essential for cell membrane repair. **(A)** Membrane repair assay performed on human control myoblasts (WT, C25), dysferlin-null human myoblasts (DYSF-null, AB320) and transfected dysferlin-null myoblasts (DYSF-null + mCherry-dysferlin1, DYSF-null + mCherry-dysferlin11, DYSF-null + mCherry-dysferlin1 + mCherry-dysferlin11) in the presence of Ca2+ and membrane-impermeable dye FM 1-43. Fluorescence images were taken every second for 5 min. The damage was induced at t = 10 s. Scale bar, 10 µM. White arrows indicate the injury site. **(B)** Summary data for membrane repair assay. Black arrows indicate injury-time point. Data are mean ± SEM (Standard error of the mean) for *n* = 20 cells. Statistical analysis was performed using Mann-Whitney test. In order to facilitate the interpretation of the different comparisons between the conditions, the table on the right-hand side of the figure summarizes the statistical results obtained. (NS: non-significant, *: *p*-value < 0.05, **: *p*-value < 0.01).

To identify the exact location of cleavage site by calpain in dysferlin exon 40a, four plasmids containing the dysferlin transcript 11 with deletions of fragments in exon 40a were constructed: Dysf11delN, Dysf11delC, Dysf11 delN1, Dysf11delN2 (Figure 2A). Injury assays by ionomycin and cell scrapping were performed in cells and dysferlin cleavage was followed by western blot analysis with Hamlet-1 antibody (Figure 3B). Protein extracted from injured cells transfected with dysferlin transcript 11 revealed the presence of the mini-dysferlin band as the expected size whereas, in injured cells transfected with dysferlin transcript 1 no signal for the mini-dysferlin was detected, only the full-length dysferlin band was observed. This observation was in accordance with the calpain cleavage site located in exon 40a.

Then, exon 40a was deleted in two parts (deletion of the N-terminal side - DelN and the C- terminal side—DelC), In injured cells transfected with dysferlin delN no signal at the predicted dysferlin cleaved size was observed, while injured cells transfected with the delC construct a weak signal was detected. Thus, the N-terminal part would be more important than the C-terminal part for the recognition and cleavage of this region by calpains.

Furthermore, injured cells transfected with dysferlin delN1 (deletion of exon 40a first seven residues) and delN2 (deletion of exon 40a second quarter) constructs presented a weaker mini-dysferlin band compared to injured cells transfected with dysferlin transcript 11-construct (Figure 2B). Moreover, when we created substitutions and small deletions in exon 40a we were still able to detect dysferlin cleavage. Surprisingly, none of the mutations tested abolishes the cleavage of this domain by calpains, as presented in Supplementary Table S1. In addition, we were able to demonstrate that cleavage in exon 40a of DYSF is mediated by calpains 1 and/or 2. Indeed, cell scratch injury of the cell in the presence of a cocktail of calpain 1 or 2 inhibitors prevents the cleavage of exon 40a of DYSF (Supplementary Figure S1). Altogether these experiments demonstrate that dysferlin is cleaved in exon40a by calpains (1 and/or 2). Furthermore, the deletions made within exon 40a indicate that calpains recognize a sequence towards the N-terminal part of the exon. This minimal sequence is composed of the first 11 amino acids of the polypeptide encoded by exon 40a.

### Genetics Analysis of Dysferlin Exon 40a Sequence

Sequence analysis, in August 2021, of exon 40a using Kaviar and gnomAD databases, revealed 23 single nucleotide polymorphisms with variant frequency lower than 1.12 10^−4^ (Table 2). Interestingly, only four missense SNPs were predicted pathogenic by UMD-Predictor: c.4698G > A, c.4704C > T, c.4707G > T and c.4742A > C.

**TABLE 2:** Exon 40a variant referenced in gnomAD and Kaviar.

Variant		Position (GRCh38)	Annotation	UMD-Predictor	Allele frequency
c.4693A > C	p.Ala1472Ala	71620552	Synonymous	Polymorphism	6,39E-06
c.4696C > T	p.Asp1473Asp	71620555	Synonymous	Polymorphism	6,38E-05
c.4697delG	p.Gly1474ValfsTer33	71620555	Frameshift	—	1,06E-05
c.4697G > A	p.Gly1474Ser	71620556	Missense	Probable polymorphism	1,12E-04
c.4698G > A	p.Gly1474Asp	71620557	Missense	Pathogenic	6,39E-06
c.4702G > A	p.Leu1475Leu	71620561	Synonymous	Polymorphism	1,92E-05
c.4703T > C	p.Ser1476Pro	71620562	Missense	Polymorphism	3,19E-05
c.4704C > T	p.Ser1476Leu	71620563	Missense	Pathogenic	6,92E-05
c.4705G > A	p.Ser1476Ser	71620564	Synonymous	Polymorphism	—
c.4707G > T	p.Ser1477Ile	71620566	Missense	Pathogenic	—
c.4712G > C	p.Ala1479Pro	71620571	Missense	Polymorphism	6,39E-06
c.4717C > T	p.Pro1480Pro	71620576	Synonymous	Polymorphism	1,92E-05
c.4717C > G	p.Pro1480Pro	71620576	Synonymous	Polymorphism	1,06E-05
c.4721delAC	p.Thr1483GlyfsTer9	71620580	Frameshift	—	6,39E-06
c.4725C > T	p.Thr1583Met	71620584	Missense	Polymorphism	3,83E-04
c.4726G > A	p.Thr1583Thr	71620585	Synonymous	Polymorphism	1,28E-04
c.4730T > A	p.Ser1585Thr	71620589	Missense	Polymorphism	1,28E-05
c.4731C > T	p.Ser1585Phe	71620590	Missense	Probably pathogenic	1,06E-05
c.4733C > T	p.Pro1586Ser	71620592	Missense	Polymorphism	1,28E-05
c.4737C > T	p.Pro1587Leu	71620596	Missense	Probably pathogenic	6,39E-06
c.4741C > T	p.Ser1588Ser	71620600	Synonymous	Polymorphism	6,39E-06
c.4742A > C	p.Ser1589Arg	71620601	Missense	Pathogenic	1,28E-06
c.4745C > T	p.Pro1590Ser	71620604	Missense	Probable polymorphism	6,39E-06

### Dysferlin Transcript 11 is Essential for Membrane Repair

Given the functional importance of the cleavage site encoded by exon 40a, the dysferlin transcript 11 seems to be essential for membrane repair mechanism. To demonstrate this, we performed a membrane repair assay on control human myoblasts (WT), dysferlin-null human myoblasts (DYSF-null) and transfected dysferlin-null human myoblasts (DYSF-null + mCherry-dysferlin1, DYSF-null + mCherry-dysferlin11 and DYSF-null + mCherry-dysferlin1 + mCherry-dysferlin11) in presence of calcium and membrane-impermeable dye FM 1-43 (Figure 3). Membrane injuries on control myoblasts lead to an FM dye entry that ceases within a minute after injury whereas it continued for at least 5 min in dysferlin-null myoblasts. When dysferlin transcript 1 is expressed in dysferlin-null myoblasts, we observed a robust FM dye entry after injury nevertheless, it is slowing down after a few minutes. On the other hand, injured dysferlin-null myoblasts expressing dysferlin transcript 11 showed a slight FM dye entry that cease within a minute, comparable to what was observed for control myoblasts. A significant difference (*p* < 0.01) was obtained between untransfected dysferlin-null myoblasts and dysferlin-null myoblasts transfected with dysferlin transcript 11. Similar results were obtained when dysferlin-null myoblasts expressed both transcripts 1 and 11. These results indicated that dysferlin transcript 11 is essential in the cell membrane repair when cell undergoes laser-induced injury.

### Dysferlin Transcript 11 Participates in the Protection of Membrane Myoblasts From Mechanical Stress induced by Osmotic Shock

**FIGURE 4:**
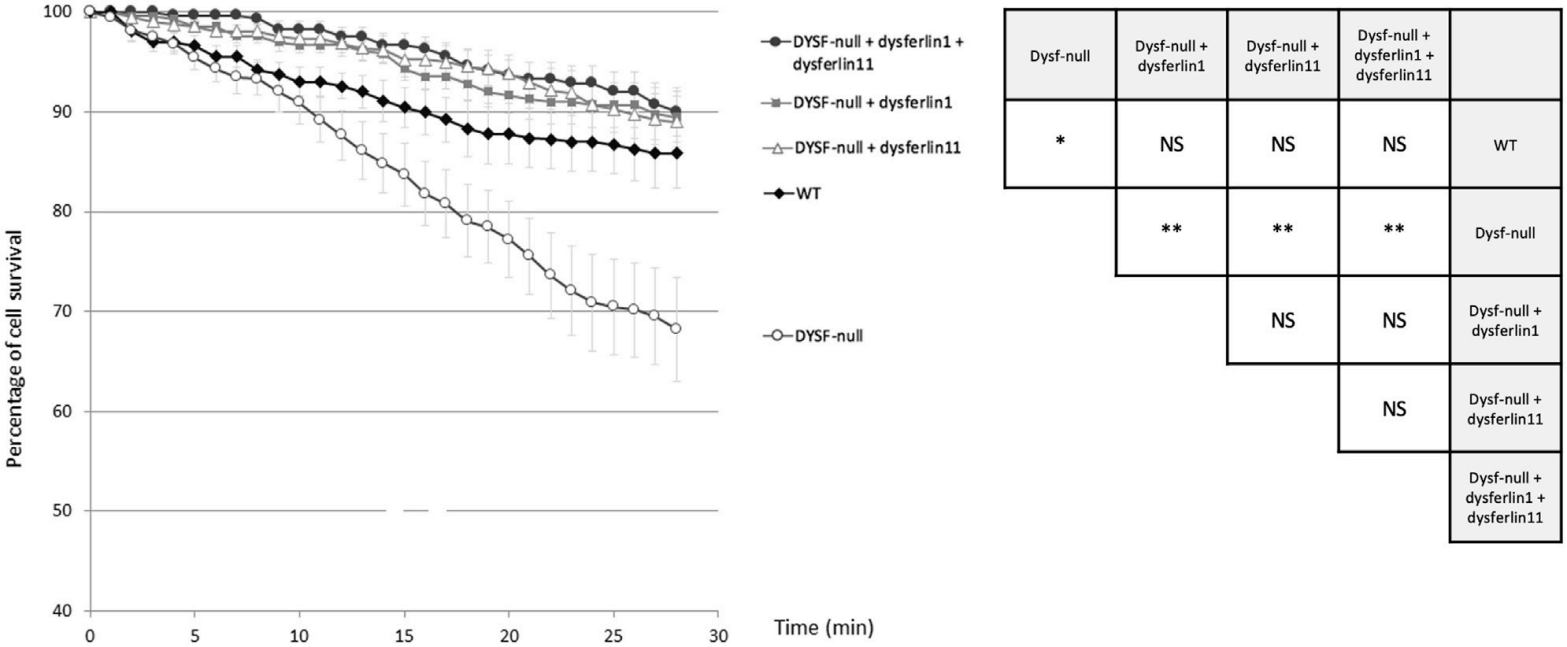
Dysferlin 11 transcript participates in the protection of membrane myoblasts from mechanical stress induced by osmotic shock. Osmotic shock assay performed on control human myoblasts (WT, C25), dysferlin-null human myoblasts (DYSF-null, AB320) and transfected dysferlin-null human myoblasts (DYSF-null + GFP-dysferlin1, DYSF-null + GFP-dysferlin11, DYSF-null + GFP-dysferlin1 + GFP-dysferlin11) in hypotonic solution (ultrapure distilled water). Fluorescence images were taken every minute for 30 min. Medium change was done at t = 1 min. All data are mean ± SEM (Standard error of the mean) for *n* = 10. For each experiment, at least 20 cells were observed. Statistical analysis was performed using Mann-Whitney test (NS: non-significant, *: *p*-value < 0.05, **: *p*-value < 0.01).

To explore the role of dysferlin transcript 11 in the protection of membrane myoblasts, we performed an osmotic shock assay on control human myoblasts (WT), dysferlin-null human myoblasts (DYSF-null) and transfected dysferlin-null human myoblasts (DYSF-null + GFP-dysferlin1, DYSF-null + GFP-dysferlin11 and DYSF-null + GFP-dysferlin1 + GFP-dysferlin11) in hypotonic solution (Figure 4). Hypo-osmotic shock led to cell swelling which induces mechanical stress on the cell membrane. To monitor the integrity of the membrane, we observed GFP fluorescence that leaks out of the cells when the plasma membrane is damaged. The percentage of cell survival for control myoblasts during hypo-osmotic shock decreased slightly during the first 15 minutes but stabilized after that (86% of cell survival percentage after 20 min of hypo-osmotic shock). Conversely, hypo-osmotic shock on myoblasts lacking dysferlin induced a decrease in the percentage of cell survival which is not stabilized after 20 min (68% of cell survival percentage after 20 min of hypo-osmotic shock). When dysferlin transcript 1 or dysferlin transcript 11 were expressed into dysferlin-null myoblasts, we can observe a slight decrease in the percentage of cell survival upon hypo-osmotic shock, as for control myoblasts (90% of cell survival percentage after 20 min of hypo-osmotic shock). These observations showed that dysferlin transcript 1 and dysferlin transcript 11 protect myoblasts from mechanical stress induced by osmotic shock.

### Dysferlin Transcript 11 Participates in Protein Vesicle Trafficking

**FIGURE 5:**
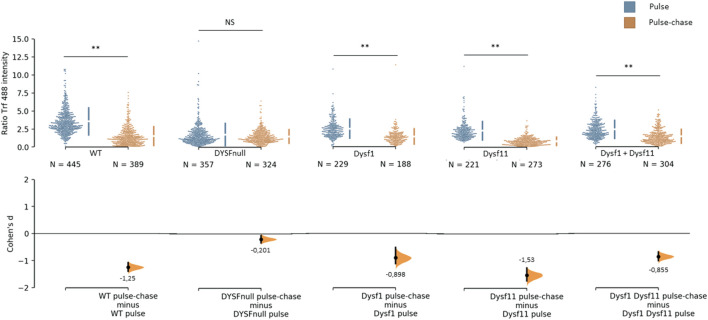
Dysferlin 11 transcript participates in protein vesicle trafficking. Transferrin assay performed on control human myoblasts (WT, C25), dysferlin-null human myoblasts (DYSF-null, AB320) and transfected dysferlin-null human myoblasts (DYSF-null + mCherry-dysferlin1, DYSF-null + mCherry-dysferlin11, DYSF-null + mCherry-dysferlin1 + mCherry-dysferlin11). Myoblasts were incubated for 30 min with transferrin coupled with Alexa Fluor 488 (pulse) and for 30 min with transferrin-free medium (pulse-chase). For each condition, transferrin intensity was measured in more than 180 cells observed in three independent experiments. Statistical analysis was performed using Mann-Whitney test (NS: non-significant, *: *p*-value < 0.05, **: *p*-value < 0.01) and the Cohen’s coefficient is presented below each graph.

It has been demonstrated that dysferlin is involved in protein vesicle trafficking. To explore the role of dysferlin transcript 11 in protein vesicle trafficking, we performed a transferrin assay on control human myoblasts (WT), dysferlin-null human myoblasts (DYSF-null) and transfected dysferlin-null human myoblasts (DYSF-null + mCherry-dysferlin1, DYSF-null + mCherry-dysferlin11 and DYSF-null + mCherry-dysferlin1 + mCherry-dysferlin11) (Figure 5). Transferrin is a molecule that is rapidly internalized into cells via the transferrin receptor. After internalization, transferrin is recycled to the plasma membrane. To explore the endocytosis function, myoblasts were incubated for 30 min with transferrin coupled to the fluorophore Alexa-488. Next, we evaluated the recycling pathway by incubating myoblasts for 30 min with transferrin and then incubating myoblasts for 30 min with a transferrin-free medium. For each cell, we quantified the intensity of transferrin. Endocytosis function (pulse) compared with recycling pathway (pulse-chase) shows a significant difference for control myoblasts in contrast with dysferlin-null myoblasts. Indeed, the Cohen’s coefficient (d; measuring the effect size) was larger for control myoblasts (−1.25) than for dysferlin-null myoblasts (−0.2). Similarly, transfected dysferlin-null myoblasts with transcript 1 and/or transcript 11 also show a significant difference between endocytosis (pulse) and the recycling pathway (pulse-chase), which demonstrates an efficient transferrin trafficking in those cells. These observations indicate that both dysferlin transcript 1 and dysferlin transcript 11 participate in protein vesicle trafficking in cells.

## Discussion

This study demonstrates the importance of the dysferlin transcript 11 which contains the alternative exon 40a. Indeed, membrane injuries induce the establishment of a repair mechanism that triggers dysferlin cleavage by calpains within exon 40a, releasing a C-terminal fragment named mini-dysferlin or mini-dysferlin C72.

Cell scratch injuries and western blot analysis performed in this study showed that cleavage in DYSF exon 40a is carried out by calpains 1 and/or 2. These results are consistent with previous studies (Mellgren et al., 2009). Several studies outline the predictions of calpain cleavage, but there is no apparent explicit rule for calpain specificity (Cuerrier et al., 2005; Shinkai-Ouchi et al., 2016). Amino acid sequences around calpains cleavage sites are very diverse. It is therefore difficult to precisely determine the location of calpains cleavage sites. Our results demonstrate that partial deletion of exon 40a decreases calpain cleavage (delC, delN1 and delN2) and deletion of exon 40a first half prevent calpain cleavage (delN). Therefore, the entire sequence of exon 40a is involved in the cleavage of dysferlin by calpains but the N-terminal part of this alternative exon seems the most important.

Furthermore, our results show that substitutions and small deletions in exon 40a of DYSF do not prevent cleavage by calpains. It was observed, for instance, that substitution or deletion of up to seven amino acids did not prevent dysferlin cleavage by calpains. Only the deletion of the twelve first amino acids of DYSF exon 40a avoids cleavage of dysferlin. These observations confirm that calpains recognize a large amino acid sequence to cleave dysferlin.

Interestingly, myoferlin, which is a member of the Ferlin protein family, is also cleaved by calpains within an alternative exon, releasing a C-terminal fragment that has 62.82% amino acid identity with the mini-dysferlin (Piper et al., 2017). There seems to be an evolutionary preservation of enzymatic cleavage of both dysferlin and myoferlin. Indeed, DYSF alternative exon 40a is conserved in most mammals.

Given the importance of the region coded by DYSF exon 40a (site of dysferlin cleavage by calpains during repair of the membrane), dysferlin transcript 11 must have a key role in the functions of muscles. Our results indicated that this transcript is essential for the repair of the membrane. The main transcript of dysferlin (transcript 1) seems to be less involved in the repair mechanism after a laser injury. These results are in line with the work of Lek and her colleagues in 2013 that showed dysferlin cleavage at exon 40a by calpains and accumulation of mini-dysferlin at lesion site during repair of the muscle cell membrane.

Noteworthy, dysferlin is also involved in other cell functions. In 2013, Kerr and colleagues showed that dysferlin-deficient muscle has a lower resistance to osmotic shock (Kerr et al., 2013). Our results confirm this previous work: dysferlin-null myoblasts are more prone to die during hypo-osmotic shock, unlike control myoblasts. Our results also show that restoration of dysferlin transcript 1 and/or dysferlin transcript 11 appeared to increase the resistance of the cells to mechanical stress induced by osmotic shock. This observation indicates that the alternative exon 40a was involved in membrane protection, but was not essential, as dysferlin transcript 1 was also capable of protecting the cell.

Moreover, dysferlin is involved in protein vesicle trafficking. Demonbreun and colleagues demonstrated in 2011 that dysferlin-null myoblasts have abnormal vesicular trafficking compared to control myoblasts (Demonbreun et al., 2011). They showed that dysferlin-null myoblasts accumulate transferrin, and that transferrin endocytic recycling is delayed in these cells. Although our results are different from theirs, we show equally an abnormal vesicular trafficking in dysferlin-null myoblasts compared to control myoblasts and dysferlin-null myoblasts with a dysferlin restoration. Our work demonstrates that dysferlin-null myoblasts have a lower transferrin accumulation than control myoblasts, and no significant difference between endocytosis function (pulse) and recycling pathway (pulse-chase) was observed. When dysferlin transcript 1 and/or dysferlin transcript 11 are restored in dysferlin-null myoblasts, we detected a significant difference between endocytosis and recycling pathway which demonstrate a restoration of vesicular trafficking. These two dysferlin transcripts seem to participate in transferrin trafficking in cells. This observation indicates, as before, that alternative exon 40a is dispensable for protein vesicle trafficking, but dysferlin exon 40a presence in transcript 11 is not interfering in this pathway.

In parallel, exon 40a genetics analysis using databases revealed 23 single nucleotide polymorphisms with low frequencies. Among these SNPs, only four missense polymorphisms were predicted pathogenic by UMD-Predictor: c.4698G > A, c.4704C > T, c.4707G > T and c.4742A > C. Therefore, we carried out constructions in which we induced a substitution of the amino acids concerned for three of these four SNPs predicted pathogenic. Western blot analysis performed after cell scratch injury with these two constructions showed that amino acid substitutions at these positions were not able to block the cleavage of dysferlin by calpains during the repair mechanism. Furthermore, our results indicate that substitutions and small deletions in exon 40a of DYSF cannot prevent calpain cleavage and mini-dysferlin creation. Knowing this, we assume that most missense variants in the alternative exon 40a may not lead to membrane repair defects. Remarkably, to date, no report has been published with mutations in dysferlin exon 40a associated with a muscular dystrophy presentation. In contrast to the expression level in muscle, transcripts containing exon 40a are more highly expressed in other tissues. The levels found in the liver, placenta, lung and pancreas vary between 40 and 60% of total dysferlin transcripts (Redpath et al., 2014). In these tissues, the presence of mutations in exon 40a could be more deleterious and lead to diseases with non-muscle symptoms. Accordingly, the presence of four predicted pathogenic variants in databases, even if they have not been validated by functional tests, supports our belief that pathogenic mutations in this exon may exist.

All these results indicate that dysferlin transcript 1 and dysferlin transcript 11 have an important role in myocyte homeostasis. In the context of therapies, these results suggest that the dysferlin transcript containing exon 40a could be restored in patients affected with dysferlinopathies. Currently, the therapeutic studies conducted only restored the main transcript of dysferlin (transcript 1) (Lostal et al., 2010; Sondergaard et al., 2015; Potter et al., 2018). These preclinical studies show promising perspectives: high levels of dysferlin expression, improvement of the histological aspect of the muscle, reduction of necrotic fibers and global improvement in locomotor activity. However, with respect to muscle membrane repair function, the improvements obtained may not restore repair to the same level as in controls.

## Conclusion

In conclusion, the data obtained demonstrate the importance of the dysferlin transcript 11, which contains the alternative exon 40a, in the myocyte membrane repair mechanism. Our results show that dysferlin transcript 11 also participates in cell membrane protection and protein vesicle trafficking. All these results indicate that dysferlin transcript 11 has an important role in the functions of the myocyte. From a therapeutic perspective, these findings support the need to restore the dysferlin transcript containing the alternative exon 40a in patients with dysferlinopathies.

## Data Availability

The original contributions presented in the study are included in the article/Supplementary Material, further inquiries can be directed to the corresponding author.

## Abbreviations

GFP, Green Fluorescent Protein; hEGF, human Epidermal Growth Factor; hFGF, human Fibroblast Growth Factor; LGMD, Limb-Girdle Muscular Dystrophy; MM, Miyoshi Myopathy; WT, Wild Type.
