# Supplementary figures and images for: The Dysferlin Transcript Containing the Alternative Exon 40a is Essential for Myocyte Functions
Source: Front Cell Dev Biol. 2021 Nov 23;9:754555. doi: 10.3389/fcell.2021.754555 (PMC8650162; doi:10.3389/fcell.2021.754555)

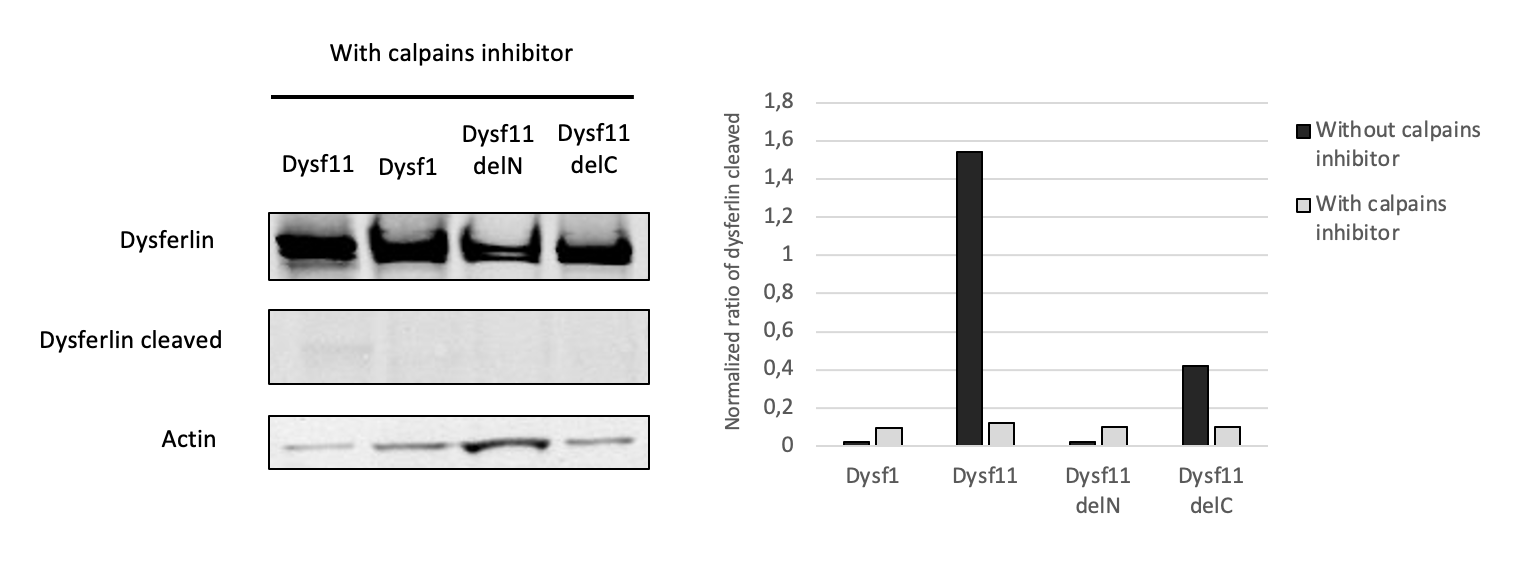

Supplement: Supplementary file 1 [file Image1.PNG]
