# Supplementary material for: The Dysferlin Transcript Containing the Alternative Exon 40a is Essential for Myocyte Functions
Source: Front Cell Dev Biol. 2021 Nov 23;9:754555. doi: 10.3389/fcell.2021.754555 (PMC8650162; doi:10.3389/fcell.2021.754555)
Supplement: Supplementary file 2 [file DataSheet1.docx]

Supplementary Material

# Supplementary Figures

## Supplementary Table 1. Substitutions and small deletions in DYSF exon 40a were not able to prevent calpain cleavage. Summary of dysferlin11 constructs build to identify calpain cleavage site in DYSF exon 40a by cell scrape injury and Western Blot analysis.

| ID | Exon 40a modification | Calpain cleavage |
| --- | --- | --- |
| S1485A  P1487A | CTTGCAGACGGTCTGTCGAGCTTGGCCCCCACTAACACGGCTGCTCCTGCATCCAGTCCTCAT | Yes |
| S1485A  P1487A  S1489G | CTTGCAGACGGTCTGTCGAGCTTGGCCCCCACTAACACGGCTGCTCCTGCATCCGGTCCTCAT | Yes |
| S1476Y | CTTGCAGACGGTCTGTACAGCTTGGCCCCCACTAACACGGCTTCTCCTCCATCCAGTCCTCAT | Yes |
| S1476A  S1477G | CTTGCAGACGGTCTGGCGGGCTTGGCCCCCACTAACACGGCTTCTCCTCCATCCAGTCCTCAT | Yes |
| T1481A | CTTGCAGACGGTCTGTCGAGCTTGGCCCCCGCTAACACGGCTTCTCCTCCATCCAGTCCTCAT | Yes |
| T1483K | CTTGCAGACGGTCTGTCGAGCTTGGCCCCCACTAACAAGGCTTCTCCTCCATCCAGTCCTCAT | Yes |
| S1488Y | CTTGCAGACGGTCTGTCGAGCTTGGCCCCCACTAACACGGCTTCTCCTCCAACCAGTCCTCAT | Yes |
| Del PTNT | CTTGCAGACGGTCTGTCGAGCTTGGCC-------------------------GCTTCTCCTCCATCCAGTCCTCAT | Yes |
| Del P1480 | CTTGCAGACGGTCTGTCGAGCTTGGCC------ACTAACACGGCTTCTCCTCCATCCAGTCCTCAT | Yes |
| P1480S | CTTGCAGACGGTCTGTCGAGCTTGGCCCTCACTAACACGGCTTCTCCTCCATCCAGTCCTCAT | Yes |
| A1472Y | CTTTATGACGGTCTGTCGAGCTTGGCCCCCACTAACACGGCTTCTCCTCCATCCAGTCCTCAT | Yes |
| Del LAD | ------------------GGTCTGTCGAGCTTGGCCCCCACTAACACGGCTTCTCCTCCATCCAGTCCTCAT | Yes |

**Figure Legend**

**Supplementary Figure 1. Calpain inhibitors 1 and 2 prevent calpain cleavage in exon 40a of DYSF. Western Blot analysis performed on** proteins extracted from HEK cells, transfected with dysferlin constructs as indicated. Twenty-four hours post-transfection cells were injured by cell scraping and ionomycin, in the presence of a calpain inhibitor cocktail for calpains 1 and 2. Hamlet-1 was used to detect the full-length dysferlin and the cleaved dysferlin. Actin was used for normalization.

The graph on the right shows the ratio of the amount of cleaved dysferlin to the amount of complete dysferlin in the presence or absence of the calpain inhibitors.
